# Cold-active pectinolytic activity produced by filamentous fungi associated with Antarctic marine sponges

**DOI:** 10.1186/s40659-018-0177-4

**Published:** 2018-08-27

**Authors:** Gabriela Poveda, Carlos Gil-Durán, Inmaculada Vaca, Gloria Levicán, Renato Chávez

**Affiliations:** 1 Departamento de Biología, Facultad de Química y Biología, Universidad de Santiago de Chile (USACH), Alameda 3363, Estación Central, 9170022 Santiago, Chile; 2 Departamento de Química, Facultad de Ciencias, Universidad de Chile, Las Palmeras 3425, Ñuñoa, Santiago, Chile

**Keywords:** Cold-active pectinases, Antarctic marine sponges, Filamentous fungi, *Geomyces* sp.

## Abstract

**Background:**

Pectinase enzymes catalyze the breakdown of pectin, a key component of the plant cell wall. At industrial level, pectinases are used in diverse applications, especially in food-processing industry. Currently, most of the industrial pectinases have optimal activity at mesophilic temperatures. On the contrary, very little is known about the pectinolytic activities from organisms from cold climates such as Antarctica. In this work, 27 filamentous fungi isolated from marine sponges collected in King George Island, Antarctica, were screened as new source of cold-active pectinases.

**Results:**

In semi-quantitative plate assays, 8 out 27 of these isolates showed pectinolytic activities at 15 °C and one of them, *Geomyces* sp. strain F09-T3-2, showed the highest production of pectinases in liquid medium containing pectin as sole carbon source. More interesting, *Geomyces* sp. F09-T3-2 showed optimal pectinolytic activity at 30 °C, 10 °C under the temperature of currently available commercial mesophilic pectinases.

**Conclusion:**

Filamentous fungi associated with Antarctic marine sponges are a promising source of pectinolytic activity. In particular, pectinases from *Geomyces* sp. F09-T3-2 may be potentially suitable for biotechnological applications needing cold-active pectinases. To the best of our knowledge, this is the first report describing the production of pectinolytic activity from filamentous fungi from any environment in Antarctica.

## Background

Among the macromolecules that compose the plant cell wall, pectin is one of the most abundant and complex. Pectin is a family of diverse polysaccharides that comprise, at least, seven structural elements, being homogalacturonan, xylogalacturonan, rhamnogalacturonan I and rhamnogalacturonan II the most widely known [1, 2]. From a chemical point of view, pectin is composed by a main chain of galacturonic acid residues bound by β (1 → 4) linkages (homogalacturonan), or by a mix of galacturonic acid and rhamnose (rhamnogalacturonans) or galacturonic acid and xylose (xylogalacturonan). In turn, the main chain can be substituted by a variety of molecules, such as methyl, ethyl, and diverse sugar moieties (arabinose, rhamnose, galactose, and others) [3].

According to its complex structure, biodegradation of pectin requires a pool of several enzymes, collectively named as pectinases. These pectinases include pectin methyl esterases, pectin acetyl esterases, polygalacturonases, polymethylgalacturonases, polygalacturonate lyases, polymethylgalacturonate lyases, rhamnogalacturonase, arabinases and xylogalacturonases [2].

Pectinases have great biotechnological potential, mainly in the food industry. Pectinases are used to remove the suspended pectin from raw juices in fruit juices processing, thus avoiding the increased viscosity that inabilities the filtering process. In winemaking, in addition to the improvement of mash filtering, pectinases can be also used to improve the juice extraction from the grapes and to release compounds responsible for the color and aroma in wines [4, 5].

Among the microorganisms able to degrade pectin, the filamentous fungi are among the most efficient. They have demonstrated a great capability of secreting a wide range of pectin-degrading enzymes, and currently, most of the commercial pectinolytic enzymes available are produced by filamentous fungi, particularly from genera *Aspergillus*, *Trichoderma* and *Penicillium* [1, 6, 7].

By far, most of the commercial pectinases are of mesophilic origin, and they account up to 40% of the enzymes used in food industry [2]. These mesophilic commercial pectinases have optimal temperatures between 40 and 60 °C [2]. However, there are processes where pectin degradation is necessary at lower temperatures. For example, the clarification of the mash for the production of white wine and *pisco* is performed at 15 °C. This low temperature is required to avoid the propagation of microbiota and to keep intact the aromatic molecules, which confer the organoleptic characteristics to these products. Recent investigations indicate that commercial pectinases with mesophilic characteristics do not work efficiently during wine fermentations at low temperatures [8]. Thus, in the last years the interest to seek cold-active pectinases (with optimal temperatures below 40 °C) is increasing. These cold-active pectinases potentially could replace the existing mesophilic commercial enzymes in low-temperature processes. Microorganisms isolated from cold regions of the Earth are able to produce cold-active pectinases, and to date, several yeasts and some bacteria with this ability have been isolated from samples of Argentinian Patagonia, Himalayan regions, Iceland and Japan [2]. On the contrary, the information about filamentous fungi producing cold-active pectinases is rather scarce. Although in literature there are several papers claiming for the production of cold-active pectinases or cold-active pectinolytic activities by filamentous fungi (both from mesophilic and cold-loving fungi), almost all of them report the production of pectinolytic enzymes with optimal activities at 40–45 °C [9–14]. Thus, to the best of our knowledge, pectinases from filamentous fungi with optimal activity lower than 40 °C have been identified only in *Botrytis cinerea* [14].

Antartica is one of the most pristine, remote and cold regions in the Earth. Thus, this place seems suitable for the prospection of new microorganisms producing cold-active enzymes, including pectinases. Bacteria and yeasts able to degrade pectin have been isolated from different Antarctic environments [16–19], but remarkably, to the best of our knowledge, there are no studies reporting the successful production of cold-active pectinases from filamentous fungi isolated from any environment in Antarctica.

Recently, we have obtained cultivable filamentous fungi from Antarctic marine sponge samples [20]. We hypothesize that these fungi could be producers of cold-active enzymes, including pectinolytic activity. Therefore, the objective of this study was to evaluate if these Antarctic filamentous fungi can produce cold-active pectinases.

## Methods

### Fungal strains

The fungal strains used in this work are described in Table 1. All of them were previously obtained from Antarctic marine sponges [20] and belong mostly to *Geomyces* sp. and *Pseudogymnoascus* sp., which are recognized cold-loving organisms [21]. Most of them are unidentified species (see “Discussion”). All the fungal strains were routinely kept on potato dextrose agar (PDA) until use.

**Table 1:** Fungal strains isolated from Antarctic marine sponges that were used in this wok

Strain^a^	Genus or species^b^
F09-T1-5	*Pseudogymnoascus pannorum*
F09-T1-10	*Pseudogymnoascus pannorum*
F09-T3-1	*Geomyces* sp.
F09-T3-2	*Geomyces* sp.
F09-T3-4	*Geomyces* sp.
F09-T3-5	*Geomyces* sp.
F09-T3-8	*Geomyces* sp.
F09-T3-17	*Geomyces* sp.
F09-T7-1	*Penicillium polonicum*
F09-T7-2	*Penicillium polonicum*
F09-T9-1	*Penicillium* sp.
F09-T10-1	*Penicillium* sp.
F09-T12-1	*Cladosporium* sp.
F09-T12-2	*Cladosporium* sp.
F09-T13-12	*Geomyces* sp.
F09-T14-2	*Thelebolus* sp.
F09-T15-6	*Epicoccum* sp.
F09-T16-1	*Phoma* sp.
F09-T18-1	*Pseudogymnoascus* sp.
F09-T18-12	*Pseudogymnoascus* sp.
F09-T18-14	*Pseudogymnoascus* sp.
F09-T18-15	*Pseudogymnoascus* sp.
F09-T18-16	*Pseudogymnoascus* sp.
F09-T18-19	*Pseudogymnoascus* sp.
F09-T18-20	*Pseudogymnoascus* sp.
F09-T18-23	*Pseudogymnoascus* sp.
F09-T23-3	*Acremonium* sp.

^a^Strain nomenclature according to Henriquez et al. [20]

^b^Identity of the strains according to Henriquez et al. [20]

### Screening of pectinolytic activity

In preliminary experiments, we observed that fungi associated with Antarctic marine sponges have optimal temperature for growth at 15 °C (data not shown), so we used this temperature in all the experiments. At that temperature, pectinolytic activity was screened upon inoculation of fungal isolates on agar plates containing Czapek-agar plus pectin as the sole carbon source (NaNO_3_ 10 g/L, K_2_HPO_4_ 2 g/L; MgSO_4_⋅7 H_2_O 0.5 g/L, FeSO_4_⋅7H_2_O 0.01 g/L, pectin from citrus peel (Sigma) 10 g/L, agar–agar 2%; pH adjusted at 5.5 with NaOH). Fungi were grown during 7 days in triplicate, and pectinolytic activity was determined by staining the plates with 1% cetyltrimethyl ammonium bromide (CTAB) solution. CTAB has the ability to precipitate acid polysaccharides in solution, so it is commonly used to detect pectinolytic activity on agar plates [22–24]. Briefly, 5 mL of CTAB solution was added to each plate and incubated during 30 min. After that, excess of CTAB solution was eliminated, and the enzymatic activity index (EAI) was calculated as the halo/colony diameter (h/c) ratio according to de García et al. [25]. EAI is a semi-quantitative parameter commonly used to quickly estimate the enzymatic activity of microorganisms grown on solid media [26, 27].

### Production of pectinolytic activity in liquid medium

Flasks containing 100 mL of liquid Czapek-pectin medium (the same composition as above, without agar–agar) were inoculated with 1 × 10^7^ spores, and incubated at 15 °C and 180 r.p.m. during 10 days. Supernatant samples were withdrawn daily, and pectinolytic activity was measured as described below.

### Quantitative pectinolytic activity assays

For pectinase activity measurement, each reaction mix contained 200 µL sodium acetate buffer 500 mM pH 5.5, 200 µL pectin solution [pectin from citrus peel (Sigma) 0.5%, pH 5.5] and 25 µL of the suitable supernatant sample. The reaction mix was incubated during 30 min at 37 °C. Reaction was stopped by addition of 640 µL of dinitrosalicylic acid solution (dinitrosalicylic acid 1%, sodium potassium tartrate 30% and NaOH 1.6%) and incubation at 95 °C for 5 min. At these conditions, dinitrosalicylic acid reacts with the reducing sugar released from pectin, producing a complex with maximal absorbance at 540 nm. Thus, the reaction was then cooled in ice by 5 min, and centrifuged to obtain the supernatant. Absorbance of the supernatant was measured at 540 nm, and absorbance data were interpolated in a suitable calibration plot. The pectinolytic activity (U/mL) was calculated as the enzyme necessary to release 1 µmol of reducing sugars for minute. Specific activity (U/mg) was obtained normalizing the activity by protein concentration, determined by the Bradford’s method [28].

To determinate the effect of temperature on pectinolytic activity, the same assay described above was performed, but at different temperatures. For details of temperatures used, see the respective Figure.

## Results

### Screening of pectinolytic activity in fungi from Antarctic marine sponges

**Fig. 1:**
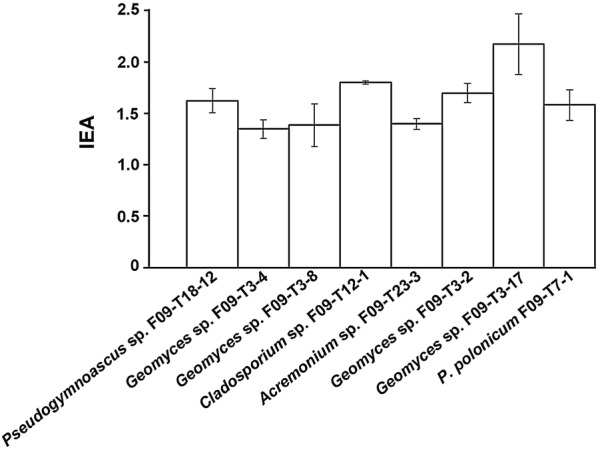
Pectinolytic activity of Antarctic fungi from marine sponges on agar plates. Pectinolytic activity expressed as EAI was calculated as the halo/colony diameter (h/c) ratio. Only those isolates whose EAI was higher than 1.0 are shown. The name and code of each fungal isolate is indicated under each bar. Temperature of assay was 15 °C. Each experiment was done in triplicate, and error bars indicate the SD of mean values. Differences in pectinolytic activity detected were not statistically significant (p < 0.05 using Student’s-t test)

Nineteen out 27 strains grew on Czapek-pectin medium, but did not show halo of degradation in the plate assay at 15 °C (data not shown), suggesting that they have poor pectinolytic activity at low temperature. The rest of the isolates (eight fungi) showed different degree of intensity of the pectinolytic activity by the halo/colony ratio (Fig. 1). All these fungi had similar behavior, with EAI around 1.5–2.0 (Fig. 1). These eight fungi were used to estimate their production of pectinolytic activity in liquid medium (see below).

### *Geomyces* sp. F09-T3-2 produces the highest levels of pectinolytic activity in liquid medium

**Fig. 2:**
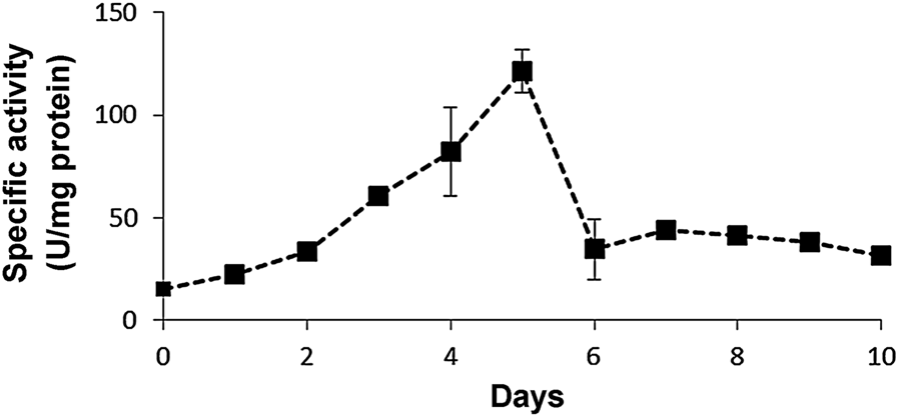
Production of pectinolytic activity by *Geomyces* sp. F09-T3-2. *Geomyces* sp. F09-T3-2 was grown in liquid cultures containing pectin as the sole carbon source at 15 °C. Supernatants samples were withdrawn daily and pectinolytic activity was measured according the quantitative assay described in “Methods”. Each measurement was done in triplicate, and error bars indicate the SD of mean values of specific activity

The eight fungi showed in Fig. 1 were used to quantitatively estimate the production of pectinolytic activity in liquid medium at 15 °C during 10 days. By far, the strain *Geomyces* sp. F09-T3-2 produced the highest levels of pectinolytic activity. At day 5, this strain produced maximal specific activity (121 U/mg; Fig. 2). The rest of the strains tested produced barely detectable activity compared with this strain (data not shown), so they were discarded for further experiments. Differences in activities observed between plate assays and measurements in liquid medium can be explained by different conditions used in these assays. Plate assays are semi-quantitative and give a rough idea of the potential of the fungi as producers of cold-adapted pectinases. The assay takes 7 days, and during this time, pectinases secreted diffuse through the agar gel, degrading pectin. Thus, the result observed in plate assay corresponds to enzymes acting on pectin during several days. On the contrary, the measurement of specific activity is performed in liquid medium, and compared to plate assay, it takes a very short time (30 min). The differences can be also explained by the sensitivity of the assays. In plate assay, similar halos can be produced by few but highly active enzymes, or by much enzyme with low activity. On the contrary, specific activity can discriminate both situations. Thus, two fungi can give similar patterns in plate assay, but their specific activities can be very different because different enzymatic conversion rates, or differences in the total protein produced by each fungus.

### *Geomyces* sp. F09-T3-2 shows optimal pectinolytic activity at 30 °C

**Fig. 3:**
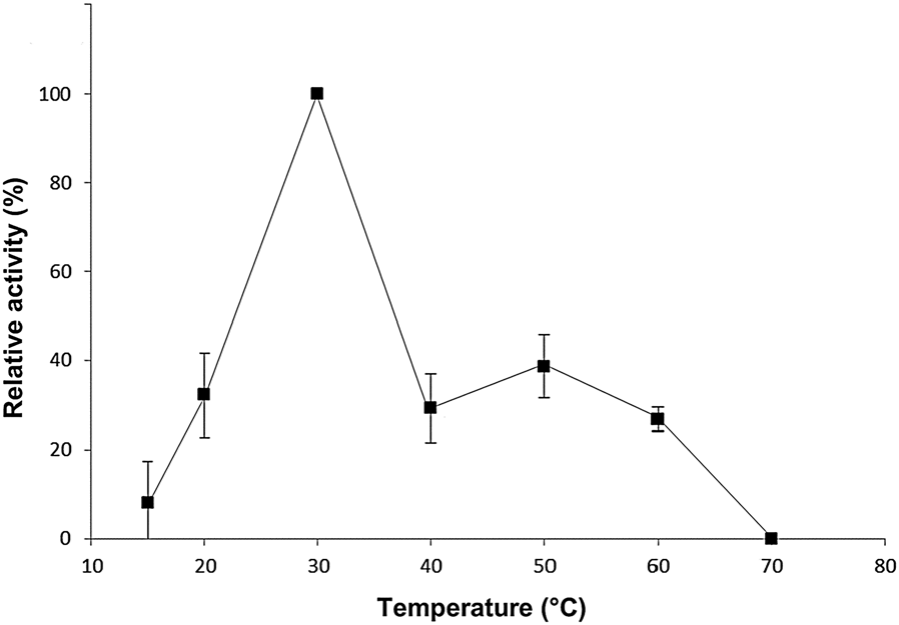
Effect of temperature on pectinolytic activity of *Geomyces* sp. F09-T3-2. Supernatant samples obtained at day 5 (day of maximal production, see Fig. 2) were used. Pectinolytic activity was measured according the quantitative assay described in “Methods”, except that temperature of assay was varied. Maximal specific activity obtained at 30 °C was set as 100% activity, and the average specific activities obtained at other temperatures were normalized and expressed as percentage with respect to activity at 30 °C. Values are expressed as mean ± standard deviation of three independent readings

We tested the performance of pectinolytic activity of *Geomyces* sp. F09-T3-2 at different temperatures (Fig. 3). We found that pectinases of this strain have a good performance at low temperatures. Our results indicate that optimal temperature for activity of pectinases from *Geomyces* sp. F09-T3-2 is 30 °C, which is 10 degrees lower than those observed for the commercial pectinases from mesophilic fungus (see “Discussion”). In fact, to the best of our knowledge, these results suggest that pectinases from *Geomyces* sp. F09-T3-2 have the lowest optimal temperature among the fungal pectinases described so far (see “Discussion”).

## Discussion

Pectins are a heterogeneous group of polysaccharides that compose the plant cell wall. In food industry, high amounts of pectin are released during processing of fruits, which tend to remain in the suspension, resulting in an increase in viscosity and turbidity, which hampers the clarification process. This problem is usually solved by the use of pectinolytic enzymes [29]. Commercial pectinases have optimal activity temperatures between 40 and 60 °C, but some processes (such as white wine and *pisco* production) occur at lower temperatures. Thus, pectinases working at lower temperatures are necessary. Here we have identified a fungal strain with optimal pectinolytic activity at 30 °C, at least 10 °C lower than most of the fungal pectinases described so far. This strain would be an ideal candidate for future purification of cold-active pectinases.

To the best of our knowledge, there are no reports describing the successful production of pectinolytic activity from filamentous fungi isolated from Antarctica. Loperena et al. [30] characterized the production of pectinolytic activity in several Antarctic fungi using a similar plate semi-quantitative analysis, but they did not found any filamentous fungi producing pectinolytic activity. On the contrary, we found eight strains (representing 30% of the Antarctic filamentous fungi analyzed in this work) producing pectinolytic activity (Fig. 1). Thus, this may be the first result demonstrating the production of pectinolytic activity in filamentous fungi from any Antarctic origin.

Our strains producing pectinolytic activity includes four strains of *Geomyces* sp., one strain of *Pseudogymnoascus* sp., one strain of *Acremonium* sp., one strain of *Cladosporium* sp. and one strain of *P. polonicum*. Pectinolytic activity has been already described in *Penicillium*, *Cladosporium* and *Acremonium* species [31–35], but not in *Geomyces* or *Pseudogymnoascus* species. Thus, pectinolytic activity in these fungal genera is reported here for the first time. *Geomyces* sp. and *Pseudogymnoascus* sp. are saprophytic cold-loving fungi [21] commonly found in cold environments including marine and terrestrial Antarctica [20, 30, 36, 37]. *Geomyces* sp. and *Pseudogymnoascus* sp. are allied (phylogenetically closer) genera whose taxonomical placement was recently re-evaluated [38]. As a result, currently just one species of *Geomyces* (*G. auratum*) is formally recognized [38]. Interestingly, and according to a preliminary analysis (data not shown), none of the *Geomyces* sp. strains that showed pectinolytic activity in Fig. 1 (including the best producer *Geomyces* sp. F09-T3-2) belong to *G. auratum*, so they would be new species waiting for a formal taxonomical description. Regarding *Pseudogymnoascus* sp., it is a very diverse and extent group of species, most of them undescribed yet, whose taxonomic relationships are not totally clear [38].

The production of pectinase activity under 40 °C by filamentous fungi is rare. This is true even in psychrophilic and psychrotolerant filamentous fungi. For example, *Sclerotinia borealis*, a pathogenic fungus found in regions extremely cold that does not grow at temperatures higher than 20 °C, produces pectinases with optimum activity at 40 °C [9]. Another case is *Mucor flavus*, a psychrotolerant fungus with optimal growth at 15 °C that produces pectinases with optimal activity at 45 °C [10]. Thus, to the best of our knowledge, in the literature there is only one example of a filamentous fungus producing pectinases with optimum activity below 40 °C. This belongs to the phytopathogenic fungus *Botrytis cinerea*, which produces pectinases with optimal activities between 34 and 37 °C [15]. In our case, we observed that the optimal temperature of the pectinolytic activity of *Geomyces* sp. F09-T3-2 was 30 °C (Fig. 3). Thus, the pectinases from *Geomyces* sp. F09-T3-2 may have the lower optimal temperature described so far for any pectinase from filamentous fungi, making this strain a promissory candidate for the purification of cold-active pectinases with potential biotechnological applications.

## Conclusion

To the best of our knowledge, this work is the first describing the production of pectinolytic activity in any Antarctic filamentous fungi. Our results suggest that filamentous fungi associated with Antarctic marine sponges are potential producer of pectinases. In particular, the isolate *Geomyces* sp. F09-T3-2 showed optimal pectinolytic activity at 30 °C, the lower temperature described so far for this activity in any filamentous fungus. Thus, pectinases from this isolate may be potentially suitable for biotechnological applications such as clarification of mash for the production of white wine and *pisco*.

## Abbreviations

PDA: potato dextrose agar
CTAB: cetyltrimethyl ammonium bromide
EAI: enzymatic activity index
r.p.m.: revolutions per minute

## References

[1] Benoit I, Coutinho PM, Schols HA, Gerlach JP, Henrissat B, de Vries RP (2012). Degradation of different pectins by fungi: correlations and contrasts between the pectinolytic enzyme sets identified in genomes and the growth on pectins of different origin. BMC Genomics.

[2] Adapa V, Ramya LN, Pulicherla KK, Rao KR (2014). Cold active pectinases: advancing the food industry to the next generation. Appl Biochem Biotechnol.

[3] Willats WGT, McCartney L, Mackie W, Knox JP (2001). Pectin: cell biology and prospects for functional analysis. Plant Mol Biol.

[4] Zoecklein BW, Marcy JE, Williams JM, Jasinsky Y (1997). Effect of native yeasts and selected strains of *Saccharomyces cerevisiae* on glycosyl, glucose potential, olatile terpenes and selected aglycones of white Riesling (*Vitis vinifera*) wines. J Food Compos Anal.

[5] Ganga A, Piñaga F, Querol A, Vallés S, Ramón D (2001). Cell-wall degrading enzymes in the release of grape aroma precursors. Food Sci Technol Int.

[6] Gupta R, Kalpana (2011). Optimization of production and reaction conditions of polygalacturonase from *Byssochlamys fulva*. Acta Microbiol Immunol Hung..

[7] Lara-Márquez A, Zavala-Páramo MG, López-Romero E, Cano Camacho H (2011). Biotechnological potential of pectinolytic complexes of fungi. Biotechnol Lett.

[8] Reynolds AG, Knox A, Di Profio F (2018). Evaluation of macerating pectinase enzyme activity under various temperature, pH and ethanol regimes. Beverages..

[9] Takasawa T, Sagisaka K, Yagi K, Uchiyama K, Aoki A, Takaoka K (1997). Polygalacturonase isolated from the culture of the psychrophilic fungus *Sclerotinia borealis*. Can J Microbiol.

[10] Gadre RV, Van Driessche G, Van Beeumen J, Bhat MK (2003). Purification, characterisation and mode of action of an endo-polygalacturonase from the psychrophilic fungus *Mucor flavus*. Enzyme Microb Technol.

[11] Saito K, Takakuwa N, Oda Y (2004). Purification of the extracellular pectinolytic enzyme from the fungus *Rhizopus oryzae* NBRC 4707. Microbiol Res.

[12] Niture SK, Pant A (2004). Purification and biochemical characterization of polygalacturonase II produced in semi-solid medium by a strain of *Fusarium moniliforme*. Microbiol Res.

[13] Dinu D, Nechifor MT, Stoian G, Costache M, Dinischiotu A (2007). Enzymes with new biochemical properties in the pectinolytic complex produced by *Aspergillus niger* MIUG 16. J Biotechnol.

[14] Pan X, Tu T, Wang L, Luo H, Ma R, Shi P (2014). A novel low-temperature-active pectin methylesterase from *Penicillium chrysogenum* F46 with high efficiency in fruit firming. Food Chem.

[15] Johnston DJ, Williamson B (1992). Purification and characterization of four polygalacturonases from *Botrytis cinerea*. Mycol Res.

[16] Truong LV, Tuyen H, Helmke E, Binh LT, Schweder T (2001). Cloning of two pectate lyase genes from the marine Antarctic bacterium *Pseudoalteromonas haloplanktis* strain ANT/505 and characterization of the enzymes. Extremophiles..

[17] Vaz ABM, Rosa LH, Vieira MLA, de Garcia V, Brandão LR, Teixeira LCRS (2011). The diversity, extracellular enzymatic activities and photoprotective compounds of yeasts isolated in Antarctica. Braz J Microbiol..

[18] Carrasco M, Rozas JM, Barahona S, Alcaíno J, Cifuentes V, Baeza M (2012). Diversity and extracellular enzymatic activities of yeasts isolated from King George Island, the sub-Antarctic region. BMC Microbiol.

[19] Tropeano M, Coria S, Turjanski A, Cicero D, Bercovich A, MacCormack W (2012). Culturable heterotrophic bacteria from Potter Cove, Antarctica, and their hydrolytic enzymes production. Polar Res.

[20] Henríquez M, Vergara K, Norambuena J, Beiza A, Maza F, Ubilla P (2013). Diversity of cultivable fungi associated with Antarctic marine sponges and screening for their antimicrobial, antitumoral and antioxidant potential. World J Microbiol Biotechnol.

[21] Hayes MA (2012). The Geomyces fungi: ecology and distribution. Bioscience.

[22] da Silva EG, de Fátima Borges M, Medina C, Piccoli RH, Schwan RF (2005). Pectinolytic enzymes secreted by yeasts from tropical fruits. FEMS Yeast Res.

[23] Wang L, Stegemann JP (2010). Extraction of high quality RNA from polysaccharide matrices using cetlytrimethylammonium bromide. Biomaterials.

[24] Zhao L, Xu Y, Lai XH, Shan C, Deng Z, Ji Y (2015). Screening and characterization of endophytic *Bacillus* and *Paenibacillus* strains from medicinal plant *Lonicera japonica* for use as potential plant growth promoters. Braz J Microbiol..

[25] de García V, Brizzio S, Libkind D, Buzzini P, van Broock M (2007). Biodiversity of cold-adapted yeasts from glacial meltwater rivers in Patagonia, Argentina. FEMS Microbiol Ecol..

[26] Goldbeck R, Andrade C, Pereira G, Filho M (2012). Screening and identification of cellulase producing yeast-like microorganisms from Brazilian biomes. Afr J Biotechnol..

[27] Taskin E, Eltem R, Simplício da Silva E, Braga de Souza JV (2008). Screening of *Aspergillus* strains isolated from vineyards for pectinase production. J Food Agric Environ.

[28] Bradford MM (1976). A rapid and sensitive method for the quantitation of microgram quantities of protein utilizing the principle of protein-dye binding. Anal Biochem.

[29] Docco T, Williams P, Cheynier V (2007). Effect of flash release and pectinolytic enzyme treatments on wine polysaccharide composition. J Agric Food Chem.

[30] Loperena L, Soria V, Varela H, Lupo S, Bergalli A, Guigou M (2012). Extracellular enzymes produced by microorganisms isolated from maritime Antarctica. World J Microbiol Biotechnol.

[31] Barbosa MAG, Rehn KG, Menezes M, de Mariano R (2001). LR. Antagonism of *Trichoderma* species on *Cladosporium herbarum* and their enzymatic characterization. Braz J Microbiol..

[32] Gomes E, Ribeiro Leite RS, da Silva R, Silva D (2009). Purification of an exopolygalacturonase from *Penicillium viridicatum* RFC3 produced in submerged fermentation. Int J Microbiol..

[33] Jurick WM, Vico I, McEvoy JL, Whitaker BD, Janisiewicz W, Conway WS (2012). *Penicillium solitum* produces a polygalacturonase isozyme in decayed Anjou pear fruit capable of macerating host tissue in vitro. Mycologia.

[34] Bastos SC, Pimenta CJ, Dias DR, Chalfoun SM, Angélico CL, Tavares LS (2013). Pectinases from a new strain of *Cladosporium cladosporioides* (Fres.) De Vries isolated from coffee bean. World J Agric Sci..

[35] Gao M-T, Yano S, Minowa T (2014). Characteristics of enzymes from *Acremonium cellulolyticus* strains and their utilization in the saccharification of potato pulp. Biochem Eng J..

[36] Loque CP, Medeiros AO, Pellizzari FM, Oliveira EC, Rosa CA, Rosa LH (2010). Fungal community associated with marine macroalgae from Antarctica. Polar Biol.

[37] Goncalves VN, Vaz AB, Rosa CA, Rosa LH (2012). Diversity and distribution of fungal communities in lakes of Antarctica. FEMS Microbiol Ecol.

[38] Minnis AM, Lindner DL (2013). Phylogenetic evaluation of *Geomyces* and allies reveals no close relatives of *Pseudogymnoascus destructans*, comb. nov., in bat hibernacula of eastern North America. Fungal Biol..

